# A First Look at Childhood Abuse in Women with Obstructive Sleep Apnea

**DOI:** 10.21203/rs.3.rs-2842895/v1

**Published:** 2023-05-03

**Authors:** Amrita Pal, Fernando Martinez, Jennifer Wagman, Ravi S. Aysola, Ari Shechter, Vincent Mysliwiec, Jennifer Martin, Paul M. Macey

## Abstract

**Study objectives.** Women who experienced childhood sexual abuse have higher rates of obesity, a risk factor for obstructive sleep apnea (OSA). We assessed if prior childhood sexual abuse was more common in women with OSA vs. control, with possible mediation by obesity. **Methods** . We studied 21 women with OSA (age mean±s.d. 59±12 years, body mass index (BMI) 33±8 kg/m ^2^ , respiratory event index [REI] 25±16 events/hour, Epworth Sleepiness Scale [ESS] 8±5) and 21 women without OSA (age 53±9 years, BMI 25±5 kg/m ^2^ , REI (in 7/21 women) 1±1 events/hour, ESS 5±3). We evaluated four categories of trauma (general trauma, physical, emotional, and sexual abuse) with the early trauma inventory self-report-short form (ETISR-SF). We assessed group differences in trauma scores with independent samples t-tests and multiple regressions. Parametric Sobel tests were used to model BMI as a mediator for individual trauma scores predicting OSA in women. **Results.** Early childhood sexual abuse reported on the ETISR-SF was 2.4 times more common in women with vs. without OSA ( *p* =0.02 for group difference). Other trauma scores were not significantly different between women with and without OSA. However, BMI was a significant mediator ( *p* =0.02) in predicting OSA in women who experienced childhood physical abuse. **Conclusions.** Childhood sexual abuse was more common in a group of women with OSA than those without OSA. Additionally, BMI was a mediator for OSA of childhood physical but not sexual abuse. There may be physiological impacts of childhood trauma in women that predispose them to OSA.

## Introduction

Though less common in women than men, an estimated 17% of adult females have obstructive sleep apnea (OSA) [1], and there may be sex-specific factors contributing to this occurrence. In particular, childhood sexual abuse is experienced more frequently by women than men and can increase the risk of developing many sleep disorders [2–9]. Given obesity is also another well-established risk factor for OSA [10], a possible underlying mechanism could be childhood trauma-associated weight gain [11], also common in women [11–16]. The link between childhood sexual abuse and obesity is well established, as is the obesity-OSA connection; the question is whether childhood sexual trauma is an independent risk factor for OSA.

The mechanisms by which obesity leads to OSA are well-studied [17]. Obesity leads to fat deposition in the upper airway and resulting metabolic activity of adipose tissue may promote inflammatory cytokines that may increase pharyngeal collapsibility through mechanical effects on pharyngeal soft tissues and lung volume and altered signaling of airway neuromuscular nerves [18, 19]. Muscle tone may be reduced due to damage to the genioglossus nerve from mitochondrial dysfunction elicited by an obesity-related diet [20, 21]. Such pathophysiology can produce alterations in the mechanical and neural control of upper airway collapsibility, which determines sleep apnea susceptibility [22].

The objective was to examine the association between childhood abuse and adult onset of OSA, based on a secondary analysis of data from an ongoing study. We focused on women given the sex-specific characteristics of sexual abuse trauma and OSA. Our hypothesis was that female sexual abuse survivors would have a higher rate of OSA than females without a history of sexual abuse. We also hypothesized that BMI would mediate any relationship between trauma and OSA.

## Methods

For this secondary analysis, we selected 21 OSA and 21 control women participants from a larger study that was conducted between 2013 and 2022. Recruitment was in the Los Angeles area, and participants were screened to exclude control sleep and mental health disorders; major illness or traumatic brain injury; stroke; major cardiovascular disease; and recent (< 3 months) use of psychotropic medications. Participants with OSA were diagnosed with a two-night home sleep apnea test (HSAT), or in-lab overnight polysomnography (PSG) through the UCLA Sleep Disorders Center. HSAT testing was conducted with an ARES device [23], which includes electroencephalogram (EEG) measurement for sleep staging, with scoring based on the 2012 American Academy of Sleep Medicine criteria [24]. For a diagnosis of OSA, participants either had an REI ≥ 5 events/hour on HSAT or apnea-hypopnea index ≥ 5 events/hour on PSG. 42% of the OSA participants used continuous positive airway pressure (CPAP). Females control participants were selected to match the age range of the OSA group and ensure a non-significant difference in mean ages by independent samples t-test (threshold $p = 0.05$). For the control group, participants who screened positive for potential OSA based on the reported symptoms of OSA (excessive daytime sleepiness, snoring, night-time awakening out of breath) were referred for HSAT, confirming the absence of OSA. Borderline cases of OSA or control were excluded (RDI/AHI 4–6 events/hour). All participants visited UCLA, where height and weight were measured for BMI calculation, and menopausal status was recorded (categorized as pre, peri or post). They completed anxiety and trauma questionnaires in a private setting using encrypted online survey tools. All procedures were approved and performed in accordance with the relevant guidelines and regulations of the University of California Los Angeles Institutional Review Board. All participants provided written informed consent.

Trauma was measured with the early trauma inventory self-report short-form (ETISR-SF) [25, 26]. The ETISR-SF measures four categories of trauma on continuous scales: general trauma, physical abuse, emotional abuse, and sexual abuse [26]. Based on reported normative scores [26], we categorized trauma occurrence as higher than the threshold values of 1.6 for general trauma, 1.0 for physical abuse, 0.8 for emotional abuse, and 0.2 for sexual abuse [26]. Anxiety was measured with the Generalized anxiety disorder scale (GAD-7), a short self-report measure based on seven items [27].

Independent samples t-tests were run to compare trauma scores, BMI and anxiety between the OSA and control groups. We conducted a multiple regression analysis using the four sub-categories of trauma scores as predictors of OSA status. We modeled BMI mediating each of the four trauma scores on OSA diagnosis in a parametric Sobel test. The mediation test model’s coefficients were calculated using regression-based path analysis with IBM SPSS v26. The mediation test was conducted using the powerMediation R package “testMediation.Sobel” [28, 29].

## Results

The women in the OSA group (n=21) had a mean±std age of 59±9 years, BMI of 33±8 kg/m$^2$, and REI of 25±16 events/hour (Table 1). The women in the control group (n=21) had a mean age of 53±9 years, BMI of 25±5 kg/m$^2$, and REI of 1±1 events/hour in the four women who underwent HSAT. The 17 women in the control group who did not have HSAT were screened as low probability of OSA. The ESS mean score for OSA was 8±5 and control 5±3 (difference $p$=0.02). The menopausal status was distributed similarly across both groups (p = 0.8).

Sexual abuse scores were significantly ($p$=0.02) higher in women with OSA 1.62 ± 1.91, compared to women without OSA 0.48 ± 0.98 (Table 1). Sexual abuse was a significant predictor of OSA diagnosis (unstandardized regression coefficient= 0.11, standard error= 0.048, $p$=0.03) (Table 2). Other abuse scores did not show significant group differences in means or as predictors.

BMI was a significant ($p$=0.02) mediator of childhood physical abuse on OSA diagnosis. BMI was not a significant mediator of childhood sexual or emotional abuse or of general trauma on OSA diagnosis.

## Discussion

We found that a sample of women with OSA had a higher rate of childhood sexual abuse than women without OSA. While sexual abuse is not typically associated with sleep-disordered breathing, our study suggests that trauma exposure in childhood may be a risk factor for the development of OSA. Additionally, we found that BMI was a mediator of childhood physical abuse (but not sexual or other abuse) on the development of OSA in older women. BMI did not mediate the association between childhood sexual abuse and OSA, although such relationships may be identifiable in larger samples, and with more detailed assessments of childhood experiences.

Factors other than obesity that could link OSA and sexual abuse are neuroendocrine effects of aging in women, including reduced progesterone levels [30], effects which are exacerbated in sexual abuse survivors who report symptoms of trauma [16]. Brain circuitry changes may also lead to changes in breathing and cardiovascular control [31], particularly in women [8, 32]. Poor autonomic regulation including decreased heart rate variability has been found in both women with early childhood sexual abuse history [33] and OSA [34-36]; this autonomic dysfunction may extend to breathing control.

Studies by Krakow et al. [5, 37, 38] show an association with trauma and principally mild OSA, typically comorbid with insomnia [39]. Comorbid insomnia and OSA, noted by Krakow in his cohort of female sexual abuse survivors [5, 37, 38], has only recently had attention in terms of a joint diagnosis, for example as reported by Sweetman et al. and Brock et al. [40, 41]. Women who are sexual abuse or other trauma survivors may be considered more likely to have insomnia, but sleep problems may be less likely to be attributed to OSA, potentially contributing to underdiagnosis of the sleep disordered-breathing in such women [42].

Findings should be interpreted considering several limitations. This is a cross-sectional study based on secondary data analysis. The exclusion criteria of no current use of psychotropic medications meant that women with severe trauma-related anxiety needing ongoing treatment would have been excluded, thus potentially leading to a lower effect in this study than the general female OSA population. The sample size was small for mediation analysis, so the lack of a significant BMI effect is not a definitive test of the presence or absence of this pathway. Finally, the control group sleep status may not be fully accurate, since some women in the control group who were low in clinical probability for OSA did not undergo an HSAT, and a negative HSAT does not conclusively rule out OSA.

In conclusion**,** we found that a sample of women with OSA had a higher rate of childhood sexual abuse trauma compared with an age-matched healthy group. This preliminary study raises the possibility that childhood sexual abuse may place women at higher risk for developing OSA [43], but follow-up in larger samples is required to establish whether and how pathways from childhood sexual abuse trauma to OSA occur.

## Declarations

#### Acknowledgments

This research was supported by the National Institute of Nursing Research NR-017435 (PM), National Heart, Lung, and Blood Institute HL135562 (PM), Sleep Research Society Foundation Small Research Grant 2022 (AP), and VA Health Services Research and Development Services, Research Career Scientist Award RCS20-191 (JM).

#### Data availability

All data generated or analysed during this study are included in this published article [and its supplementary information file].

## Tables

#### Table 1. Participant characteristics.

A: Clinical characteristics. The REI, nadir and mean $SpO_2$ scores in control were only available in 4 of 21 women. ESS: Epworth Sleepiness Scale; REI: Respiratory Event Index. B: Percentage of women in each group with trauma, and the mean ± standard error of mean (SEM) of the trauma scores and clinical variables. P-values reflect group differences based on the chi-square text for menopause and independent samples t-tests for other variables (italicized for $p \leq 0.05$).

| **A: Clinical measures mean±SEM** | BMI kg/m$^2$ | Anxiety GAD7 | REI<br><br>events/hour | Oxygen saturation<br><br>nadir<br><br>% | Excessive daytime sleepiness<br><br>ESS | Menopause |
|---|---|---|---|---|---|---|
| C ontrol (N=21) | 25.14 ± 4.99 | 1.00 ± 1.22 | 1.00 ± 0.82 (N=4) | 89.03 ± 2.45 (N=4) | 4.71 ± 3.51 | 2 pre, 5 peri, 14 post |
| OSA (N= 21) | 33.04 ± 7.63 | 3.67 ± 3.89 | 24.65 ± 15.78 | 81.52 ± 9.11 | 8.00 ± 4.77 | 1 pre, 4 peri, 16 post |
| p-value (t test or chi-square) | *<0.001* | *0.006* | *<0.001* | *0.004* | *0.015* | 0.8 |
| | | | | | | |
| **B: ETISR-SF scores mean±SEM** | General | Physical | Emotional | Sexual | | |
| Control (N=21) | 2.62 ± 2.13 | 0.90 ± 1.34 | 1.57 ± 1.83 | 0.48 ± 0.98 | | |
| OSA (N= 21) | 2.62 ± 2.38 | 1.57 ± 1.47 | 1.81 ± 1.94 | 1.62 ± 1.91 | | |
| p-value (t test) | 0.99 | 0.13 | 0.68 | *0.021* | | |

**Table 2. Model of trauma types predicting OSA diagnosis.**

Trauma scores in the general, physical, emotional and sexual categories as predictors in a multiple regression model of OSA diagnosis (OSA or control) in women. Significant ($p \leq 0.05$) relationships are indicated in italics.

| Overall Model | Sum of Squares | df | Mean Square | F | Sig. |
|---|---|---|---|---|---|
| Regression | 2.142 | 4 | .536 | 2.371 | 0.07 |
| Residual | 8.358 | 37 | .226 | | |
| Total | 10.5 | 41 | | | |

| Model Variables | Unstandardized Coefficients | | Standardized Coefficients | t | Sig. |
|---|---|---|---|---|---|
| | B | Std. Error | Beta | | |
| Sexual | .111 | .048 | .352 | 2.296 | *0.027* |
| General | .053 | .041 | .232 | 1.291 | 0.205 |
| Physical | .128 | .076 | .362 | 1.698 | 0.098 |
| Emotional | .035 | .054 | .129 | 0.647 | 0.522 |

## Figures

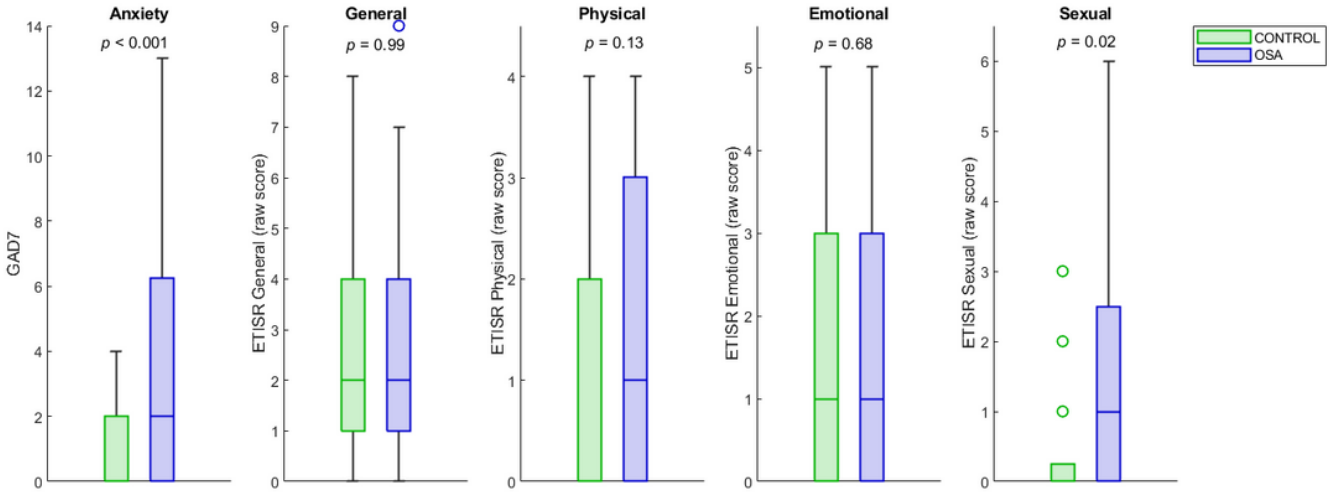

#### Figure 1

#### OSA and control mean values of childhood trauma.

The OSA and control group means and distributions of childhood sexual, physical, emotional and general abuse trauma scores are shown using box and whisker plots. The y-axes represent raw scores on the GAD7 and ETISR-SF trauma scales.

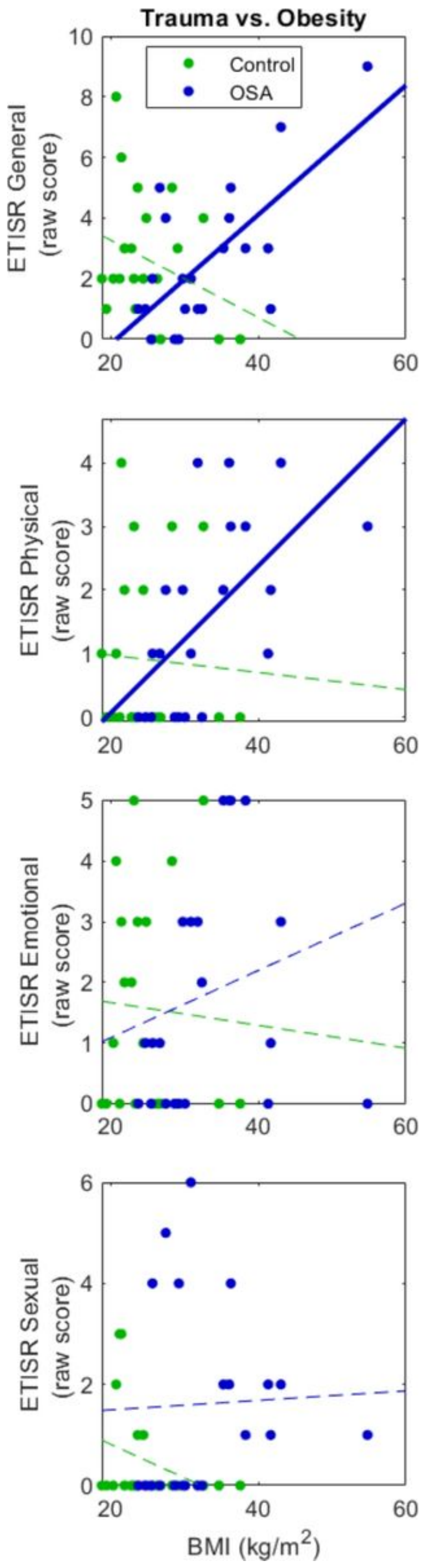

### Figure 2

**Correlations between childhood sexual trauma and BMI in OSA and control.**

The relationship between childhood trauma scores and BMI in OSA and control women are shown on scatterplots. The y-axes represent raw scores on the ETISR-SF trauma scales.

## Supplementary Files

This is a list of supplementary files associated with this preprint. Click to download.

- DataforOSAchildhoodtrauma.xlsx
